# The Moderating Role of COVID-19 Perceived Risk between Health Concern and Psychological Well-Being of Active Senior Campers Using PROCESS Macro Model

**DOI:** 10.3390/ijerph191811405

**Published:** 2022-09-10

**Authors:** Eui-Yul Choi, Mi-Hwa Jang

**Affiliations:** 1 Department of Sports Marketing, Keimyung University, Daegu 42601, Korea; 2 Department of Industry-University Cooperation, Keimyung University, Daegu 42601, Korea

**Keywords:** active senior, psychological well-being, health concern, perceived risk, COVID-19

## Abstract

This study attempts to contribute to improving the life of the elderly by empirically analyzing the factors affecting the psychological well-being of active seniors in the ‘With COVID-19’ era. To this end, the relationship between psychological well-being, health concern, and perceived risk of COVID-19 was verified for active seniors in Korea who enjoy camping. Two hundred and sixty-four valid questionnaires collected from active senior participants of “The Korea Camping Fair 2022” held in EXCO, Daegu, Korea, from 29 April to 1 May 2022, were analyzed. The main results were as follows. The health and psychological well-being of active seniors were higher than the normal level, and the perceived risk of COVID-19 was lower than the normal level. It was found that the health of active seniors had a positive effect on their psychological well-being. The perceived risk of COVID-19 was found to moderate the effect of health concern on psychological well-being. In conclusion, in order to improve the psychological well-being of active seniors, active leisure activities of the elderly are essential even in the COVID-19 situation, and various measures are needed to increase health. Not only this, but also, importantly, accurate information sharing on COVID-19 should be premised.

## 1. Introduction

The National Health Insurance Service, a Korean healthcare agency, said that the average life of Korean people is 82 years, while healthy life is 73 years, which means that many elderly people die after being sick for about 9 years. The recent pandemic caused more pain for the elderly who were experiencing difficult old age, due to physical aging and loss of social roles [1]. As of August 2021, the Korea Disease Control and Prevention Agency said 20.6% of all confirmed cases were made up of senior citizens aged 60 or older, and, especially, their mortality rate reached 93.99% [2]. As such, the elderly experienced a high risk of infection and had a 12 times higher risk of death due to the underlying disease and the atypical nature of COVID-19 [3,4].

A life of coexistence with the virus, which is still continuing, provides various risk factors to the elderly, such as deterioration of physical and mental health and restrictions on economic and social activities [1,4]. In particular, the marginalized social interaction of the elderly, due to fear of the virus and quarantine guidelines, such as social distancing, causes deterioration of physical function and worsening of chronic diseases, so it creates great fear not only for the elderly with underlying diseases, but also for the healthy elderly. Most seriously, such intense social isolation and loneliness has a negative impact on the mental health of the elderly, resulting in depression, anxiety, panic disorder, adaptation disorder, chronic stress, and insomnia [1,5,6,7]. As such, prolonged COVID-19 is having an intensely negative impact on the elderly, who account for a large proportion of the population of Korea, which has an aging society, as well as in our society as a whole. Therefore, the need to consider various dimensions to promote the healthy life of the elderly in the With-COVID-19 era is urgent.

What factors affect the psychological well-being of the elderly in the era of With-COVID-19? First of all, this study focused on seniors who are active in cultural, consumption, and physical activities based on the abundant economic power that has recently been emerging socially. Recently, among the elderly population, the baby boomers and those in their 70s and 80s who are called “active seniors” or “new seniors”, have been seen as an aging economic powerhouse [8], drawing attention in various fields of society [9]. In particular, Korean baby boomers, as key players in industrialization, are economically stable, due to their economic achievements and national pension benefits [10] and have a high level of education [11,12]. In addition, as a generation that has undergone cultural changes, they have a high awareness of, and desire for, active leisure compared to previous elderly [10], so active physical activities, such as camping, hiking, golf, etc., are preferred to passive physical activities [9,13]. According to the 2019 Camping Tourist Report of the Korea Tourism Organization [14], 27.6% of those surveyed were over 60 years old, and the figure was 29.3% in 2020, up 2% from the previous year [15]. 

Active seniors who enjoy productive leisure lives can be said to have relatively high health concerns, and this, in turn, positively affects their psychological well-being [16,17,18,19]. However, considering the coexistence of COVID-19, it can be inferred that even people with high health concerns can change their quality of life depending on whether they are aware of the negative aspects of COVID-19. In this context, this study attempted to analyze how the psychological well-being of active seniors who enjoy camping as a productive leisure activity despite the With-COVID-19 period, are affected by perceived risk of COVID-19. The study had the following detailed objectives: (1) to identify the levels of health concern, perceived risk of COVID-19, and psychological well-being of active seniors; (2) to determine whether the health concerns of active seniors affect psychological well-being; and (3) to examine whether COVID-19 risk perceived by active seniors affects the relationship between health concerns and psychological well-being.

## 2. Literature Review and Research Question Development

### 2.1. Active Senior, Camper

By 2020, Korea already had an aging society, with people aged 65 or older accounting for 15.7% of the population and baby boomers (born 1955–1963) accounting for 15.6% [20]. Korea is expected to enter a super-aged society by 2025, with a population of over 65 years old exceeding 20% [13,20]. Among the elderly population, the baby boomers and those in their 70s and 80s who ‘live young’ are attracting attention as “active seniors” or “new seniors”, due to their active consumption and leisure activities [9]. Active seniors, unlike the existing elderly, actively participate in culture and consumer life based on abundant economic power [10]. Since these active seniors’ healthy old age lives can contribute to solving various problems faced by the super-aged society and the With-COVID-19 society, various concerns relate to their health [12]. In particular, Korea’s baby boomers are highly educated [11,12], and are economically stable, due to their economic achievements and the benefits of the national pensions received as key players in Korean industrialization [10]. In addition, as a generation that has undergone cultural changes, the awareness and desire for leisure activities is very high compared to the previous elderly [10], so they prefer active physical activities, such as camping, mountain climbing, and golf, rather than passive physical activities, such as walking [9,13].

Recently, around the world, camping has been favored as an outdoor leisure activity that can be enjoyed more safely in a natural environment while avoiding the airtightness and close proximity of the With-COVID-19 era [21,22,23]. In Korea, “camping” is a hashtag that received the most attention from Instagram users in 2021 [24]. The size of the camping industry in Korea increased vertically by 90.1% to $2.353 billion in 2019 and $4.474 billion in 2020 [15], and the camping population increased about 10 times from 6 million in 2019 to 7 million in 2020 [24,25]. Camping, which was representative of nature-friendly leisure activities in the past, has been recognized as a safe and independent leisure activity, that provides ‘safe healing’ in the With-COVID-19 era, and is enjoyed as an outdoor activity that can meet the needs of tourism and leisure while observing social distancing [26,27]. As well as a safe healing leisure activity, camping was selected as a leisure activity for active seniors who preferred active physical activities in the With-COVID-19 era, and over 60s were confirmed to make up close to 30% of the camping population in Korea [15]. Therefore, this study was set for visitors aged 65 or older who attended ‘The Korea Camping Fair 2022’ to study active seniors with camping experience, camping being an active physical leisure activity.

### 2.2. Psychological Well-Being

The COVID-19 pandemic has had a huge impact on everyone, regardless of the gender, age, and gap between the rich and the poor, but it is causing the most pain to the vulnerable, especially the elderly [1]. The prolonged COVID-19 outbreak, since the first case in Korea in January 2020, has been regarded as a risk factor that has various effects on the physical and mental health and economic activities of the elderly [1,4]. Reduced social interaction due to COVID-19 leads to worsening chronic diseases and poor physical function, which is fatal not only to the elderly with underlying diseases, but also to the healthy elderly [1,28]. Especially, social isolation and loneliness negatively affect not only the physical health of the elderly, but also the social and psychological health, by causing depression, anxiety, adaptation disorder, panic disorder, insomnia, and chronic stress [1,5,6,29,30].

The well-being of the elderly in the With-COVID-19 era is very important not only personally, but also socially. Well-being was defined by the World Health Organization (WHO) in 1948 as not only being disease-free but also having psychological, spiritual, physical, and social well-being [31], classified as subjective and psychological well-being [32,33]. Well-being basically means overall satisfaction and happiness with life, but explaining well-being as positive emotions or life satisfaction is not enough. It is necessary to consider psychological health, and whether a person can function effectively as a human being [34]. Psychological well-being, which is emphasized in this respect, is an approach that focuses on positive functionalization of humans. It means not just seeking pleasure, but the degree of happiness or satisfaction that an individual feels about life from a psychologically stable and self-realizing perspective [35,36]. In other words, psychological well-being is a concept that emphasizes value, meaning, and functional dimensions, and focuses on psychologically functioning life, such as whether a person is living a life that grows autonomously with a purpose [37]. The psychological well-being of the elderly is affected by whether they are fully functioning as members of society, although out of economic activity [32]. Based on previous research, this study intended to define psychological well-being as a positive emotion that realizes the potential to change and overcome various difficult situations in the With-COVID-19 era, to pursue a sustainable life by improving the quality of life through well-being and health, and includes the concept of welfare, in terms of the abundance of life and health [35]. Therefore, people with high psychological well-being can accept themselves as they are, maintain positive interpersonal relationships, control their own behavior, control their surroundings by their own standards, have a purpose in life, and have motivation to realize their potential [38].

### 2.3. Health Concern

Health concern refers to an individual’s attitude and interest in health and healthy behaviors [39,40]. In particular, as people’s health concern increases due to COVID-19, the number of people participating in exercise to increase immunity is increasing. As such, health concern acts as a motive for efforts to improve or maintain physical and mental health and quality of life [41], and has a great influence on practicing desirable health behaviors [17,42,43]. This health concern acts as a motivation for the elderly to participate in leisure activities [39], and the higher the health concern, the higher the degree of health behavior [44,45], and good health care can overcome psychological problems caused by loss of social role [46]. As such, the elderly’s health concern in our aging society is not only about treating diseases, but is also an important motivation to seek various ways to improve health in general daily life, such as eating habits, exercise, and social activities [17].

In addition, health concern is closely related to health status and quality of life [41], and it was confirmed that it had a positive effect on the psychological well-being of the elderly. Choi and Seol [17] argued that the health concern of active seniors who enjoy golf had a positive effect on psychological well-being. Lee, Kim, and Lee [18] and Seol and Lee [19] confirmed that the health concern of active seniors had a positive effect on life satisfaction. Ahn [16] found that the more positive the perceived health status, the greater the sense of happiness. As such, it was confirmed that health concern had a psychologically positive effect, such as inducing the taking up of various leisure activities by active seniors and making them feel psychological happiness and life satisfaction. Based on the above previous studies, therefore, we set out the following research question.

Research question 1: Does health concern affect psychological well-being of active senior campers?

### 2.4. Perceived Risk of COVID-19

Perceived risk is a concept introduced by Bauer [47] at the consumption behavioral level, and is defined as an ‘uncertain risk that can cause undesirable results’, unlike objective and stochastic risks [48,49]. This is a risk that people feel before taking a specific action, and, even in the same situation, individuals may feel differently depending on their own disposition and acceptance of risk. In other words, even if there is an objective risk, perceived risk may appear different, and such differences in perception have various effects on individual attitudes and behavior [50,51,52,53,54]. Accordingly, perceived risk of COVID-19, which has recently been sweeping the world, is different among individuals, and it has been confirmed that it has various effects on individual feelings, attitudes, and behaviors. Han [55] confirmed that fear of infection with COVID-19 caused atrophy of individual leisure activities and had a negative effect on leisure satisfaction. Pakpour, Griffiths and Lin [56], in a study related to COVID-19 and psychological responses, found that negative psychological changes, such as anxiety, perceived risk, and stress, associated with COVID-19 had a significant impact on human behavior. In addition, Yıldırım and Güler [57] confirmed in a study on the relationship between perceived risk and pain and happiness regarding COVID-19, that perceived risk of COVID-19 negatively affected positivity and happiness.

In the With-COVID-19 era, therefore, even those who are health-conscious or healthy elderly people, can be negatively affected in their psychological well-being due to a perceived risk of COVID-19 [55,56,57]. As such, it can be inferred that perceived risk of COVID-19 can be a psychological moderator that must be dealt with in coexisting with the virus. Based on the previous studies that have been cited above, therefore, we set out the following research question.

Research question 2: How does perceived risk of COVID-19 affect the relationship between health concern and psychological well-being of active senior campers?

## 3. Methodology

### 3.1. Conceptual Framework

**Figure 1:**
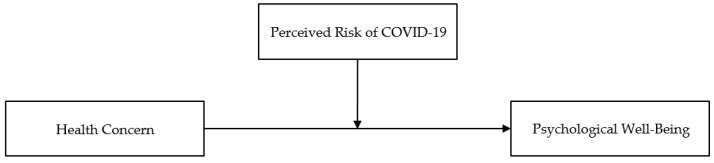
Conceptual framework of the relationship between health concern and psychological well-being, and the moderating role of perceived risk of COVID-19.

This study intended to analyze the factors affecting the psychological well-being of active seniors with camping experience in an environment of coexisting with the virus, by investigating the relationship between psychological well-being, health concern, and perceived risk of COVID-19. Based on the previous studies mentioned in the preceding chapter, the research model was set according to the procedure for analyzing the moderating effect of model 1 in PROCESS Macro Ver. 4.1 (see Figure 1).

### 3.2. Subject of Survey

This study conducted a survey of active seniors, with camping experience, aged 65 or older who attended the “The Korea Camping Fair 2022” held at EXCO in Daegu, Korea for three days from 29 April to 1 May 2022. Two trained surveyors stayed at EXCO during the period and distributed questionnaires to participants who voluntarily expressed their intention to participate. Participants in the study were fully informed about the purpose of this study and the importance of participation, and anonymity and confidentiality were ensured in completing the survey. A total of 300 questionnaires were distributed and 286 copies were collected, and 264 copies were used for the final analysis, excluding invalid questionnaires, such as insincere answers.

### 3.3. Measures

#### 3.3.1. Psychological Well-Being of Active Senior Campers

Ryff [34] measured psychological well-being in multidimensional concepts, such as self-acceptance, positive relations with others, autonomy, environmental mastery, purpose in life, and personal growth, through the scale of Psychological Well-being. Since then, various follow-up studies have modified and supplemented the scale developed by Ryff [34] to measure psychological well-being. In this study, psychological well-being items were modified and derived according to the purpose of this study, as shown in Table 1, based on the preceding studies. The four derived questionnaires were measured on a 5-point Likert scale of 1 (not at all) to 5 (very much so).

#### 3.3.2. Health Concern of Active Senior Campers

Health concern has been measured as a motivation factor, that can change the attitude or behavior of the elderly, in various previous studies, such as interest in health, health-related information acquisition, and regular checkups. Therefore, five items of health concern were adapted from the previous studies, presented in Table 2, with some modifications. The questionnaires were measured on a 5-point Likert scale of 1 (not at all) to 5 (very much so).

#### 3.3.3. COVID-19 Risk Perceived by Active Senior Campers

Several researchers have worked to develop appropriate scales for empirical research on perceived risk of COVID-19, affecting human attitude, behavior, and well-being in various ways. Yıldırım and Güler [57], in a study to develop a scale of COVID-19 perceived risk, derived items, such as Perceived likelihood of acquiring COVID-19, Worry about oneself contracting COVID-19, Worry about COVID-19 emerging as a health issue, etc., based on the risk scale for SARS presented by Brug et al. [58]. In addition, Jaspal et al. [59] derived items, such as Gut feeling of own likelihood of infection, Feeling vulnerable, Chance of getting infected, etc., from the COVID-19 Own Risk Appraisal Scale (CORAS) development study. Therefore, this study modified and derived COVID-19 perceived risk items according to the study purpose, as shown in Table 3, by using the scale modified and supplemented by Yu and Kim [60] based on the preceding studies. The five derived questionnaires were measured on a 5-point Likert scale of 1 (not at all) to 5 (very much so).

### 3.4. Data Processing

The data collected for this study was analyzed using SPSS Ver. 26.0 and PROCESS Macro Ver. 4.1 as follows. First of all, frequency analysis was conducted to understand the characteristics of active seniors, and descriptive statistics, such as mean and standard deviation, were performed to confirm their level of health concern, psychological well-being, and perceived risk of COVID-19. In addition, exploratory factor analysis and internal consistency analysis were performed to secure construct validity and reliability of the data, and correlation was analyzed to confirm the relationship and discriminant validity between the variables. After this process, the PROCESS Macro Model, proposed by Hayes [61] in regression analysis by the simultaneous input method, was employed in order to investigate the effect of health concern on psychological well-being, and confirm the moderating role of COVID-19 perceived risk. The PROCESS Macro Model has the advantage of eliminating multicollinearity by automatically providing the mean-centering function of the independent variable and moderating variable. Furthermore, the method can verify in more detail the significance of the simple slope, which is the effect of the independent variable on the outcome variable according to the moderating variable [62].

## 4. Results

### 4.1. Characteristics of Samples

Of the 264 participants, 112 were men (42.4%) and 152 were women (57.6%). In terms of camping experience, 97 people (36.7%) had less than 1 to 3 years, followed by 94 (35.6%) less than 1 year, 43 (16.3%) less than 3 to 6 years, and 30 (11.4%) more than 6 years. In change in the number of camping times before and after the COVID-19 outbreak (February 2020), “similar” was the highest with 78 people (29.5%), followed by “a little decreased” 75 (28.4%), “a little increased” 44 (16.7%), “a lot increased” 42 (15.9%), and “a lot decreased” 25 (9.5%).

### 4.2. Reliability and Validity

Exploratory factor analysis on the survey data was performed using principal component analysis with Varimax rotation method. There was no item that hindered unidimensionality or did not satisfy the factor loading of 0.5 or higher. The data consisted of three factors, namely, health concern, psychological well-being, and perceived risk of COVID-19. Each factor with an eigenvalue greater than 1 contained 4 to 5 items. Table 4 shows that these three factors explained 70.268% of total variance and a total of 14 items converged. Cronbach’s α was located between 0.852 and 0.914, indicating that the data were found to have high internal consistency [63].

### 4.3. Descriptive Statistics and Correlation Analysis

The active seniors showed similar levels of health concern (*M* = 3.801, *SD* = 0.651) and psychological well-being (*M* = 3.838, *SD* = 0.689), whereas the level of perceived risk of COVID-19 was relatively low (*M* = 2.257, *SD* = 0.739). As shown in Table 5, correlation analysis showed a positive relationship between health concern and psychological well-being and a negative relationship between perceived risk and psychological well-being. Meanwhile, in the correlation between all variables, the maximum coefficient value was 0.423, indicating that the discriminant validity between the variables was satisfied.

### 4.4. Relationship between Health Concern and Psychological Well-Being, and the Role of COVID-19 Perceived Risk

In order to verify the moderating effect of perceived risk of COVID-19 on the relationship between health concern and psychological well-being, it was analyzed according to the procedure of PROCESS Macro model 1 for SPSS proposed by Hayes [61]. Bootstrap was used for verification, the confidence interval was 95%, and the number of samples was 5000. As shown in Table 6, health concern was found to have a positive significant effect on psychological well-being (0.872, *p* < 0.001). The confidence interval of bootstrap [0.479, 1.265] did not include zero. The interaction term between health concern and perceived risk had a negative significant effect on psychological well-being (−0.167, *p* < 0.05), and the confidence interval of bootstrap [−0.317, −0.018] did not include zero. It can be seen that perceived risk of COVID-19 moderated the relationship between health concern and psychological well-being.

Previously, the moderating effect of perceived risk of COVID-19 was confirmed in the relationship between health concern and psychological well-being. The next step was to analyze the conditional effects of health concern on psychological well-being according to the level of perceived risk, and the results are shown in Table 7. The perceived risk level was given as three conditions (16th, 50th, and 84th percentiles), and the effects of health concern were all significant (*p* < 0.001). In other words, the lower the perceived risk, the higher the effect of health concern on psychological well-being.

More specifically, the significance region of the conditional effect of health concern was identified through Johnson-Neyman analysis. As shown in Table 8, the moderator value defining Johnson-Neyman significance region was 3.835, indicating that the conditional effect of health concern was significant when the perceived risk was lower than 3.835.

**Figure 2:**
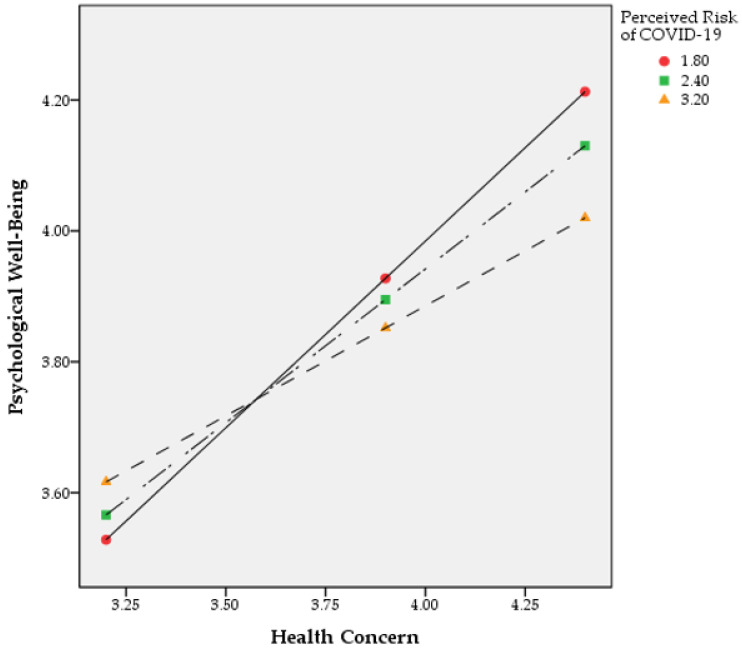
Moderating effect of COVID-19 perceived risk on the relationship between health concern and psychological well-being.

Finally, the result of visualizing the effect of health concern on psychological well-being according to perceived risk level is shown in Figure 2. In all three levels, when health concern increased, psychological well-being also increased. However, it was found that the lower the perceived risk, the steeper the slope of the increase in psychological well-being as health concern increased. In other words, it can be seen that even if the level of health concern was the same, psychological well-being further increased when the perceived risk of COVID-19 was low. However, the logic of the above interpretation is applied only when a certain degree of health concern is secured. As the health concern crossed the range of 3.500 to 3.625 (to be more precise, from 3.563), psychological well-being increased more through the moderating effect of perceived risk.

## 5. Discussion

This study attempted to contribute to improving the life of the elderly by empirically analyzing the factors affecting the psychological well-being of active seniors in the With-COVID-19 era. To this end, the relationship between psychological well-being, health concern, and perceived risk of COVID-19 was verified for active seniors in Korea who enjoy camping. Accordingly, based on the results of the study, the main implications are presented as follows.

First of all, with respect to the academic implications, as part of its consideration to improve the psychological well-being of the elderly in the With-COVID-19 era, this study focused on active seniors, an active economic and cultural consumption entity, as an aging economic powerhouse [8] that has recently attracted social attention. Active seniors in Korea are highly educated [11,12], economically stable [10], and have a high desire for leisure activities [10], preferring active physical activities like camping and hiking [9,13]. This study found more than 64% of the active seniors surveyed had enjoyed camping for more than one year despite the With-COVID-19 era, and about 62% of the elderly maintained or increased the number of camping activities even after the first case in Korea. In addition, their health concern and psychological well-being were higher than the normal level, and the perceived risk of COVID-19 was lower than the normal level. Therefore, by identifying factors that influence active seniors to age happily, we illustrate the possibility of follow-up studies for expansion into the general elderly. Second, the health concern of active seniors was found to have a positive effect on psychological well-being. This means that active seniors feel greater psychological well-being, a positive emotion that realizes their potential to change and overcome various difficult situations in the COVID-19 era, as they have more concern in health. This result is similar to the study of Choi and Seol [17] who argued that the health concern of active seniors who enjoy golf had a positive effect on psychological well-being. In addition, Lee, Kim, and Lee [18] and Seol and Lee [19] claimed that the health concern of active seniors positively affected their life satisfaction, and Ahn [16] argued that perceived health status had a positive influence on happiness. The results of these previous studies can be said to adequately support this study. As such, it was confirmed that health concern had a psychologically positive effect, such as inducing various leisure activities of active seniors and making them feel psychological happiness and life satisfaction. Third, the moderating effect of COVID-19 perceived risk was confirmed in the relationship between the health concern and psychological well-being of active seniors. That is, the lower the COVID-19 perceived risk, the higher the effect of health concern on psychological well-being. Notably here, as mentioned in the relevant section, the moderating effect is significant from the time the perceived risk is lower than the ‘existential level’ (more precisely, from the time the perceived risk is lower than 3.835). In addition, health concern must also be secured higher than the ‘normal level’ (more exactly, from the time the health interest is higher than 3.563), and then the above logic of interpretation of the moderating effect can be applied. Therefore, if active seniors with high health concern are fully aware of the role of camping, which is attracting attention as ‘safety healing’ in the With-COVID-19 era, this suggests that it can eventually have a more positive effect on their psychological well-being.

On the other hand, the practical implications of this study are as follows. First, it was confirmed that health concern not only affected various behaviors to improve the health of the elderly, but also, ultimately, had a positive effect on the psychological well-being of active seniors. Therefore, various considerations are needed to increase the health concern of the elderly in a Korean society preparing for a super-aged society, who will account for 20% of the total population. Since health concern is an important factor that can affect the physical and mental health of the elderly, such as attitude toward oneself, health promotion behavior, and psychological well-being, it is necessary to prepare measures to increase the health concern of the elderly through various channels easily accessible to them, such as mobile and senior welfare centers. Second, it is necessary to consider lowering the risk of COVID-19 perceived by active seniors. In the early days of the pandemic, vague fears stemming from ignorance of the virus restricted people psychologically as well as behaviorally. In particular, the elderly, who had a high infection rate and mortality rate, had no choice but to feel a greater risk of COVID-19. Even now, when understanding of the virus has increased and various quarantine rules are in operation, the degree of risk felt by each person is perceived differently even if it is the same virus. Since perception of this particular risk can have a significant impact on subsequent emotions, attitudes, and behaviors, various institutional arrangements are needed to help active seniors understand the virus accurately so that they can overcome their vague fear of COVID-19. Third, this study confirmed that the perceived risk of COVID-19 moderated the relationship between health concern and psychological well-being of active seniors. From the perspective of senior welfare practitioners, it is necessary to provide an optimized environment for psychological well-being of active seniors, taking into account several other unexpected variables (e.g., social, environmental, cultural, etc.) in addition to the virus pandemic these days. 

## 6. Conclusions

In this study, it was found that active seniors enjoyed active leisure activities even in pandemic situations, and their health concern and psychological well-being level were found to be higher than normal levels. In addition, it was confirmed that the perceived risk of COVID-19 was lower than the normal level. This study found that the health concern of active seniors had a positive effect on psychological well-being. The perceived risk of COVID-19 was found to moderate the effect of health concern on psychological well-being. In conclusion, for improving the psychological well-being of active seniors, active leisure activities of the elderly are essential even under COVID-19, and various measures are needed to increase health concern. Not only this, but also, importantly, accurate information sharing on COVID-19 should be premised.

Although this study has great significance in considering the psychological well-being of active seniors in With-COVID-19 era, it leaves the possibility of various follow-up studies, due to its limitations. First, this study of active seniors had academic significance, but it might be difficult to generalize because it only targeted active seniors who actively enjoy camping leisure activities in the With-COVID-19 era. Therefore, it will be necessary to expand the research subject to the general level of senior citizens who might account for about 20% of our society in the near future. Second, in addition to the variables used in this study, follow-up studies that consider in detail various demographic variables of the elderly, such as housing behavior, educational status, economic situation, and gender, will be needed.

## Figures and Tables

**Table 1:** Psychological well-being of active senior campers.

Factor	Item	Source
Psychological Well-Being	I can regulate my own behavior I can control the surrounding environment by my own standards I have a purpose in life and a motive to realize my potential I have positive interpersonal relationships	Ryff (1989) [34]; Moon et al. (2022) [38]

**Table 2:** Health concern of active senior campers.

Factor	Item	Source
Health Concern	I am very interested in health	Huang et al. (2022) [40]; Kim (2000) [44]; Ha & Choi (2013) [45]; Choi & Seol (2019) [17]; Seol & Lee (2021) [19]
	I read health-related books or watch health-related TV programs	
	I have regular health check-ups	
	I buy healthy food for my health	
	I am currently doing mental and physical training through sports for my health	

**Table 3:** COVID-19 risk perceived by active senior campers.

Factor	Item	Source
Perceived Risk of COVID-19	I have a high possibility of COVID-19 infection I am more likely to be infected with COVID-19 than other people I am likely to be easily exposed to the COVID-19 virus I may be able to come into contact with COVID-19 infected person I think other people do not follow the quarantine rules well	Yıldırım & Güler (2022) [57]; Jaspal et al. (2022) [59]; Yu and Kim (2022) [60]

**Table 4:** Exploratory factor analysis and reliability.

Factor	Item	Factor Loading			Cronbach’s α
Health Concern	HC3	**0.863**	0.119	0.026	0.889
	HC4	**0.835**	0.184	−0.001	
	HC2	**0.830**	0.166	0.077	
	HC5	**0.781**	0.221	0.028	
	HC1	**0.760**	0.170	0.024	
Psychological Well-Being	PWB2	0.207	**0.910**	−0.021	0.914
	PWB3	0.230	**0.881**	−0.023	
	PWB4	0.125	**0.875**	−0.023	
	PWB1	0.253	**0.798**	0.006	
Perceived Risk of COVID-19	PR4	0.058	−0.079	**0.833**	0.852
	PR2	−0.016	0.056	**0.826**	
	PR1	−0.054	0.033	**0.776**	
	PR5	0.091	−0.081	**0.764**	
	PR3	0.059	0.012	**0.760**	
Eigenvalue		3.511	3.177	3.150	
Variance (%)		25.077	22.690	22.501	
Cumulative Variance (%) = 70.268 Kaiser-Meyer-Olkin Measure of Sampling Adequacy = 0.818, Bartlett’s Test of Sphericity: χ^2^ = 2219.053, df = 91, *p* < 0.001					

HC = health concern; PWB = psychological well-being; PR = perceived risk of COVID-19. Factor loadings of 0.5 or higher are in bold.

**Table 5:** Correlation coefficients between variables.

Variable	Health Concern	Psychological Well-Being	Perceived Risk of COVID-19
Health Concern	1		
Psychological Well-Being	0.423 **	1	
Perceived Risk of COVID-19	0.069	−0.025	1
*M*	3.801	3.838	2.527
*SD*	0.651	0.689	0.739

** *p* < 0.01.

**Table 6:** Moderating effect of perceived risk of COVID-19.

Model	DV: Psychological Well-Being					
	Coefficient	SE	*t*	F	R^2^	ΔR^2^
Health Concern (HC)	0.872	0.199	4.369 ***	21.230 ***	0.197	0.015
Perceived Risk of COVID-19 (PR)	0.599	0.299	2.001			
HC × PR	−0.167	0.076	−2.205 *			

* *p* < 0.05, *** *p* < 0.001.

**Table 7:** Conditional effects of health concern at values of perceived risk of COVID-19.

Perceived Risk of COVID-19	Effect	SE	*t*	LLCI	ULCI
1.800	0.570	0.080	7.139 ***	0.413	0.728
2.400	0.470	0.060	7.890 ***	0.353	0.587
3.200	0.336	0.079	4.258 ***	0.181	0.491

*** *p* < 0.001; LLCI = The lower bound within the 95% confidence interval; ULCI = The upper bound within the 95% confidence interval.

**Table 8:** COVID-19 perceived risk value defining Johnson-Neyman significance region.

Perceived Risk of COVID-19	Effect	SE	*t*	LLCI	ULCI
1.000	0.704	0.129	5.463 ***	0.451	0.958
1.200	0.671	0.116	5.801 ***	0.443	0.899
1.400	0.637	0.103	6.196 ***	0.435	0.840
1.600	0.604	0.091	6.648 ***	0.425	0.783
1.800	0.570	0.080	7.139 ***	0.413	0.728
2.000	0.537	0.071	7.611 ***	0.398	0.676
2.200	0.504	0.064	7.929 ***	0.378	0.626
2.400	0.470	0.060	7.890 ***	0.353	0.587
2.600	0.437	0.059	7.351 ***	0.320	0.553
2.800	0.403	0.063	6.400 ***	0.279	0.527
3.000	0.370	0.070	5.298 ***	0.232	0.507
3.200	0.336	0.079	4.258 ***	0.181	0.491
3.400	0.303	0.090	3.372 ***	0.126	0.479
3.600	0.269	0.102	2.647 **	0.069	0.469
3.800	0.236	0.114	2.060 *	0.010	0.461
3.835	0.230	0.117	1.969	0.000	0.459
4.000	0.202	0.128	1.583	−0.049	0.453
4.200	0.169	0.141	1.193	−0.110	0.447
4.400	0.135	0.155	0.870	−0.171	0.441
4.600	0.102	0.169	0.600	−0.232	0.435
4.800	0.068	0.184	0.371	−0.294	0.430
5.000	0.035	0.198	0.175	−0.356	0.425

* *p* < 0.05, ** *p* < 0.01, *** *p* < 0.001; LLCI = The lower bound within the 95% confidence interval; ULCI = The upper bound within the 95% confidence interval.

## Data Availability

The data presented in this study are available on request from the corresponding author.
